# Is switching from brand name to generic formulations of phenobarbital associated with loss of antiepileptic efficacy?: a pharmacokinetic study with two oral formulations (Luminal® vet, Phenoleptil®) in dogs

**DOI:** 10.1186/1746-6148-9-202

**Published:** 2013-10-09

**Authors:** Marion Bankstahl, Jens P Bankstahl, Wolfgang Löscher

**Affiliations:** 1 Department of Pharmacology, Toxicology, and Pharmacy, University of Veterinary Medicine Hannover, and Center for Systems Neuroscience, Hannover 30559, Germany; 2 Present address: Preclinical Molecular Imaging, Department of Nuclear Medicine, Hannover Medical School, Hannover, Germany

**Keywords:** Epilepsy, Seizures, Antiepileptic drugs, Brand name vs. generic formulation

## Abstract

**Background:**

In human medicine, adverse outcomes associated with switching between bioequivalent brand name and generic antiepileptic drug products is a subject of concern among clinicians. In veterinary medicine, epilepsy in dogs is usually treated with phenobarbital, either with the standard brand name formulation Luminal® or the veterinary products Luminal® vet and the generic formulation Phenoleptil®. Luminal® and Luminal® vet are identical 100 mg tablet formulations, while Phenoleptil® is available in the form of 12.5 and 50 mg tablets. Following approval of Phenoleptil® for treatment of canine epilepsy, it was repeatedly reported by clinicians and dog owners that switching from Luminal® (human tablets) to Phenoleptil® in epileptic dogs, which were controlled by treatment with Luminal®, induced recurrence of seizures. In the present study, we compared bioavailability of phenobarbital after single dose administration of Luminal® vet vs. Phenoleptil® with a crossover design in 8 healthy Beagle dogs. Both drugs were administered at a dose of 100 mg/dog, resulting in 8 mg/kg phenobarbital on average.

**Results:**

Peak plasma concentrations (C_max_) following Luminal® vet vs. Phenoleptil® were about the same in most dogs (10.9 ± 0.92 vs. 10.5 ± 0.77 μg/ml), and only one dog showed noticeable lower concentrations after Phenoleptil® vs. Luminal® vet. Elimination half-life was about 50 h (50.3 ± 3.1 vs. 52.9 ± 2.8 h) without differences between the formulations. The relative bioavailability of the two products (Phenoleptil® vs. Luminal® vet.) was 0.98 ± 0.031, indicating that both formulations resulted in about the same bioavailability.

**Conclusions:**

Overall, the two formulations did not differ significantly with respect to pharmacokinetic parameters when mean group parameters were compared. Thus, the reasons for the anecdotal reports, if true, that switching from the brand to the generic formulation of phenobarbital may lead to recurrence of seizures are obviously not related to a generally lower bioavailability of the generic formulation, although single dogs may exhibit lower plasma levels after the generic formulation that could be clinically meaningful.

## Background

Epilepsy is a frequently occurring neurological brain disease in dogs [1-3]. Epilepsy in dogs can be difficult to medically control in some cases, which can lead to premature death [4]. The most common and first-choice therapy of canine epilepsy is daily oral administration of the antiepileptic drug phenobarbital, which symptomatically suppresses seizures [5-7]. Phenobarbital was introduced as a hypnotic and antiepileptic drug under the brand name Luminal® 100 years ago [8]. In order to obtain seizure control, it is recommended that plasma concentrations of phenobarbital be maintained within a therapeutic range of 10-40 μg/ml [9]. Two oral formulations of phenobarbital are approved for treatment of canine epilepsy in Europe, Phenoleptil® and Luminal® vet. Phenoleptil® is available in the form of 12.5 and 50 mg tablets, while Luminal® vet is available in the form of 100 mg tablets and, under the name Luminaletten® vet, as 15 mg tablets. Luminal® is also the trade name of the human 100 mg tablets of phenobarbital and both products (Luminal® and Luminal® vet) are produced by the same pharmaceutical company. Before Phenoleptil® and, later, Luminal® vet were approved for treatment of canine epilepsy, epileptic dogs were commonly treated with human Luminal® tablets. Following approval of Phenoleptil® for treatment of canine epilepsy, many dogs that were previously treated with human Luminal® tablets were switched to Phenoleptil®. However, it was repeatedly reported by clinicians and dog owners that switching from Luminal® (human tablets) to Phenoleptil® in epileptic dogs, which were controlled by treatment with Luminal®, induced recurrence of seizures. In humans with epilepsy, multiple studies and case reports have described relapses and worsening clinical outcome after a switch from a brand name to a generic medication [10-13]. The most likely explanation for this observation in dogs would be a lower bioavailability of phenobarbital from Phenoleptil® vs. Luminal®. To our knowledge, no bioequivalence studies on Phenoleptil® vs. Luminal® are available in dogs. Two pharmacokinetic measures, the area-under-the-curve (AUC) of the drug concentration-time curve and the maximum plasma concentration (C_max_) are used to determine bioequivalence of a generic formulation to the brand product [14]. Because the tablet formulation of Luminal® vet is identical with the standard brand name formulation Luminal®, we directly compared these pharmacokinetic measures of phenobarbital after single dose administration of Luminal® vet vs. Phenoleptil® with a crossover design in healthy Beagle dogs. Our hypothesis was that C_max_ and bioavailability of phenobarbital differ between the two pharmaceutical formulations, at least in individual dogs.

## Methods

### Animals and study design

Eight healthy Beagle dogs (2 females, 3 castrated females, and 3 castrated males) were used. They ranged in age from 1-4 years and their body weight ranged from 9.0-17.2 kg (mean 12.7 kg) at the time of the experiments. The study was designed as an open, randomized single-dose cross-over study. Experiments were performed according to the EU council directive 86/609/EWG and the German Law on Animal Protection (“Tierschutzgesetz”). Ethical approval for the study was granted by an ethical committee (according to §15 of the Tierschutzgesetz) and the government agency (Lower Saxony State Office for Consumer Protection and Food Safety; LAVES) responsible for approval of animal experiments in Lower Saxony (reference number for this project: 11A158). All efforts were made to minimize suffering of the dogs.

### Medication

Using a cross-over design, all dogs were treated with 100 mg tablets Luminal® vet (Desitin Arzneimittel; Hamburg, Germany) or 50 mg tablets Phenoleptil® (CP-Pharma, Burgdorf, Germany). Tablets were orally administered at a single dose of 100 mg phenobarbital per dog, i.e. two undivided 50 mg tablets of Phenoleptil® or one undivided 100 mg tablet of Luminal® vet per dog. Based on body weight of the individual dogs, this resulted in doses of 5.8-11.1 mg/kg (mean 7.95 mg/kg) for both formulations of phenobarbital.

Both pharmaceutical formulations were administered in an open, randomized single-dose cross-over design, i.e. four dogs were orally treated with Luminal® vet first and after a wash-out period of three weeks with Phenoleptil®. The other four dogs were treated reciprocally. Administration of tablets was performed about 24 hours after the last feeding. The dogs were fed again one hour following drug administration. The dogs had free access to water during the study.

### Blood sampling

Before and 0.5, 1, 2, 4, 6, 8, 10, 23, 32, 47, 56, 71, 80, 95, 104, and 119 hours following drug administration, venous blood samples of about 1 ml were taken. For the first eight samples, an indwelling catheter was implanted into the *vena cephalica antebrachii*. Subsequent samples were taken via single puncture of the *vena cephalica antebrachii* or *vena saphena*. Blood was collected in tubes preloaded with 20 μl 5 mM EDTA, stored on ice for a maximum of 1 hour, and centrifuged. Plasma was stored at -20°C until analysis by high performance liquid chromatography (HPLC).

### HPLC analysis

Phenobarbital concentrations in plasma samples were determined by HPLC with UV detection as described previously [15]. In short, 50 μl of plasma were mixed with 150 μl methanol, centrifuged and the supernatant injected into the HPLC apparatus equipped with a pre-column (Nucleosil 120–5 C18, 60 × 4 mm; Knauer, Berlin, Germany), a main column (Refill Nucleosil 120–5 C18, 250 × 4.6 mm; Macherey and Nagel, Düren, Germany), and an UV detector (SPD- 6A; Shimadzu, Duisburg, Germany). The mobile phase was composed of 0.05 M phosphate buffer and acetonitrile in a volume ratio of 68 to 32 (pH adjusted to 5.6). The flow rate of the mobile phase was 1 ml/min. Retention time of phenobarbital under these conditions amounted to ~6.8 min. As control, reference samples of known phenobarbital concentration were quantified every day. Limit of detection for phenobarbital was 0.2 μg/ml and limit of quantification was 0.4 μg/ml.

### Calculation of results and statistics

For statistical calculation and illustration of results, GraphPad Prism 5 (La Jolla, CA, USA) was used. Time-dependent changes in plasma concentration were modeled individually, using WinNonlin (Pharsight, St. Louis, Missouri, USA). Pharmacokinetic parameters (C_max_, peak plasma concentration; t_max_, time until C_max_ is reached; t_0.5_, elimination half-life; AUC, area under plasma concentration curves) were calculated using a single-compartment model. The relative bioavailability was calculated by dividing AUC for Phenoleptil® by the AUC of Luminal® vet. If not stated otherwise, data are expressed as mean ± standard error of the mean (SEM). Group differences were calculated by paired Student’s t-test (p < 0.05).

## Results

**Figure 1:**
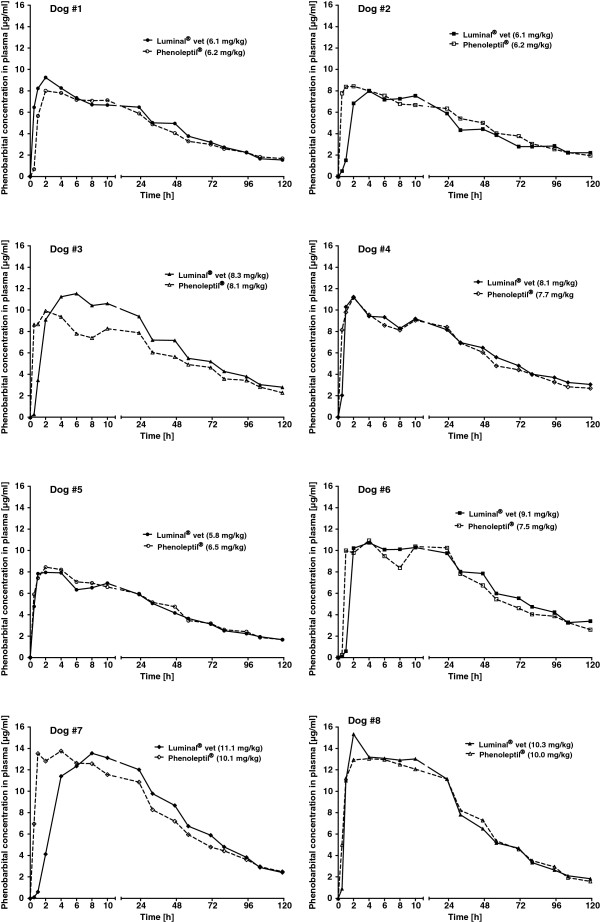
**Comparison of individual plasma concentration time curves of phenobarbital following oral administration of two different phenobarbital formulations.** Individual plasma levels after administration of Luminal® vet are shown as solid symbols and lines and those of Phenoleptil® as open symbols and hyphenated lines in 8 dogs. Each of the 8 dogs received both formulations with an interval of three weeks between the two experiments. The dose of phenobarbital was 100 mg per dog, resulting in the mg/kg doses indicated in the graphs.

**Figure 2:**
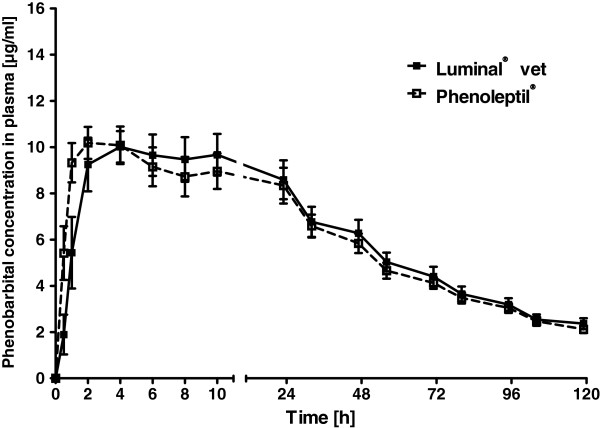
**Comparison of mean plasma concentration time curves of phenobarbital following oral administration of two different phenobarbital formulations.** Mean plasma levels after administration of Luminal® vet are shown as solid symbols and lines and those of Phenoleptil® as open symbols and hyphenated lines. Data are shown as means ± SEM of 8 dogs. See Figure 1 for individual curves.

**Figure 3:**
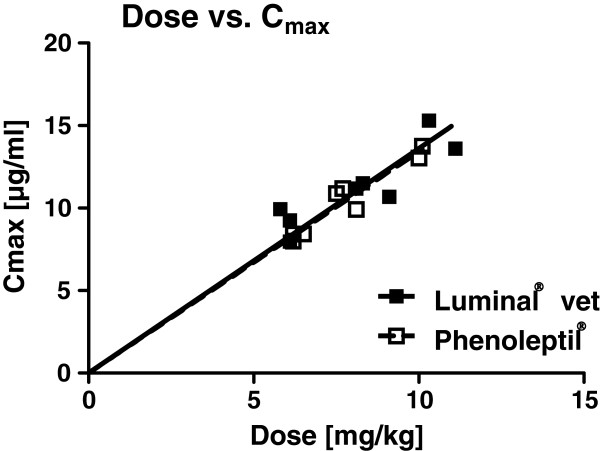
**Correlation between individual phenobarbital doses and maximal plasma concentrations.** As a result of the different body weights of the 8 dogs, doses of phenobarbital ranged between 5.8 and 11.1 mg/kg, resulting in different peak plasma levels (C_max_). As shown in the graph, a linear correlation between C_max_ and dose was obtained for both phenobarbital formulations without difference between formulations.

Individual data of phenobarbital plasma concentrations following the two formulations are shown in Figure 1, group means in Figure 2. Apart from an apparently slightly faster initial phenobarbital accumulation in blood following Phenoleptil® administration in 4 of the 8 dogs, plasma concentration curves of the two tested formulations were very similar (Figure 1). Only in dog #3, peak plasma levels of phenobarbital were clearly higher after administration of Luminal® vet. vs. Phenoleptil® (Figure 1). The variation in plasma levels from dog to dog was mainly a function of the different doses (in mg/kg) resulting from the fact that all dogs received the same overall dose (100 mg) but differed in body weight. When the observed maximal plasma concentration (C_max_; ranging from 7.95 to 15.3 μg/ml) was plotted against dose in mg/kg, a linear relationship was obtained for both formulations of phenobarbital (Figure 3).

When pharmacokinetic parameters were calculated from the plasma concentration vs. time curves shown in Figure 1, none of the parameters differed significantly between the two phenobarbital formulations. As shown in Table 1, the mean time to reach peak plasma concentrations (T_max_) tended to be higher after Luminal® vet vs. Phenoleptil® (P = 0.0588 for the calculated Tmax), but, again, no statistical significance was obtained between the two formulations. Mean peak concentration (C_max_) values in plasma were about 10 μg/ml without difference between formulations. The mean elimination half-life (t_0.5_) in plasma was about 50 h (range 37-63 h). When the relative bioavailability was calculated by dividing AUC for Phenoleptil® by the AUC of Luminal® vet, a mean value of 0.98 (range 0.90-1.15) was obtained, indicating that both formulations resulted in about the same bioavailability (Table 1). Even dog #3, which exhibited the most marked difference in peak plasma concentrations between the two formulations (Figure 1), resulting in a relative bioavailability of 0.92, stayed within the 80-125% criterion of bioequivalence [11].

**Table 1:** Pharmacokinetic parameters of phenobarbital following oral administration of 100 mg Luminal® vet or Phenoleptil® in a group of 8 dogs

**Parameter**	**Luminal® vet**	**Phenoleptil®**	**P**
T_max_ (h) observed	3.75 ± 0.79	2.75 ± 0.37	0.2275
T_max_ (h) calculated	5.97 ± 1.33	2.64 ± 0.51	0.0588
C_max_ (μg/ml) observed	10.9 ± 0.92	10.5 ± 0.77	0.2426
C_max_ (μg/ml) calculated	10.3 ± 0.92	10.0 ± 0.85	0.2569
T_0.5_ (h)	50.3 ± 3.1	52.9 ± 2.8	0.2348
AUC_(0-119)_ (μg ml^-1^ h) observed	644 ± 52.5	618 ± 44.5	0.1499
AUC∞ (μg ml^-1^ h) calculated	807 ± 66.8	781 ± 54.0	0.2961
Relative bioavailability (AUC_Phenoleptil®_ /AUC_Luminal® vet_)	0.98 ± 0.031		
	(range: 0.90-1.15; 90% CI: 0.92-1.04)		
Relative C_max_ observed (C_max, observed Phenoleptil®_/ C_max, observed Luminal® vet_)	0.96 ± 0.031		
	(range: 0.85-1.05; 90% CI: 0.90-1.03)		
Relative C_max_ calculated (C_max, calculated, Phenoleptil®)_/C_max, calculated, Luminal® vet_)	0.97 ± 0.028		
	(range: 0.81-1.08; 90% CI: 0.92-1.03)		

All data are shown as means ± SEM; for ratios also the range and 90% confidence intervals (90% CI) are shown. *Abbreviations*: T_max_, time to reach peak plasma concentration; C_max_, peak plasma concentration; t_0.5_, elimination half-life in plasma; AUC, area under the plasma concentration curve.

## Discussion

The present study had two major aims. First, to determine the pharmacokinetics of phenobarbital following oral administration of the newly licensed veterinary product Luminal® vet in dogs. The second aim of this study was to compare the bioavailability of the pharmaceutical formulations “Luminal® vet 100 mg“ (Desitin) and “Phenoleptil® 50 mg“ (CP-Pharma) following a single oral dose in dogs.

Following administration of 100 mg tablets Luminal® vet (one tablet per dog), phenobarbital was rapidly absorbed and peak plasma levels of 8-15 μg/ml were reached within 2-4 h in most dogs. Elimination was slow with half-lives ranging from 37-63 h. The data obtained from pharmacokinetic analysis of plasma concentration time curves of phenobarbital were comparable to those previously reported for Luminal® in dogs [16,17]. In the study of Al-Tahan and Frey [17], Luminal® tablets were used at a dose of 10 mg/kg, resulting in a bioavailability of 86-96% compared to i.v. administration of the same dose. Elimination half-life was 52 h after oral administration, which is almost identical with the mean half-live of 50 h determined for Luminal® vet in the present study.

A second reason to perform this study was anecdotal evidence (repeatedly reported by clinicians) that switching from Luminal® (human tablets) to Phenoleptil® in epileptic dogs, which were controlled by treatment with Luminal®, induced recurrence of seizures. The most likely explanation for this observation would be a lower bioavailability of phenobarbital from Phenoleptil® vs. Luminal®. Because the tablet formulation of Luminal® vet is identical with the formulation used in Luminal®, we directly compared bioavailability of phenobarbital after single dose administration of Luminal® vet vs. Phenoleptil® with a crossover design in healthy Beagle dogs. In contrast to our expectations, both formulations had the same relative bioavailability. Only one (#3) of the 8 dogs used for the present study showed clearly higher peak plasma levels of phenobarbital after Luminal® vet vs. Phenoleptil®. Peak plasma levels were reached more rapidly with Phenoleptil® in several dogs, which is most likely a consequence of the fact that the latter formulation was administered as two tablets of 50 mg, which may be dissolved faster in the gastrointestinal tract than the one 100 mg tablet of Luminal® vet. However, in our opinion, the resulting difference in Tmax was too small to be of any clinical relevance.

Beagles, which were used for the present study, were previously shown to eliminate phenobarbital more than 2 times more rapidly than Mongrels [16]. Such differences in the elimination rate of phenobarbital in different breeds of dogs have to be considered when treating dogs with this drug. However, we do not believe that such strain differences can explain the apparent discrepancy between the study results and the anecdotal clinical finding of poor seizure control after switching formulations.

In humans with epilepsy, loss of response after switching from brand name to bioequivalent generic formulations is a well-known phenomenon [10-12,14,18,19]. The use of pharmacokinetic bioequivalence as an indicator of therapeutic and clinical equivalence, the lack of appropriate studies comparing generic and brand name medications and differences in excipients are some of the factors that could explain variation in clinical response between generic and brand name medications [14,18,19]. Indeed, subtle differences in pharmacokinetic parameters between two formulations can produce clinically important differences in seizure control [14]. Furthermore, in case of phenobarbital, one additional reason may result from the fact that tolerance and physical dependence develops during chronic administration of this drug [20,21], so that withdrawal seizures may occur during switching from one formulation to another if a too marked decline of phenobarbital levels is not avoided during switching. In view of the consequences of increased seizure activity resulting from subtherapeutic serum drug concentration, the American Academy of Neurology (AAN) issued a position statement in 2007 opposing generic antiepileptic drug substitution without physician approval [22]. Although the present study with single doses of phenobarbital in healthy Beagle dogs did not indicate that switching from the standard brand name formulation to generic phenobarbital results in lower bioavailability in most dogs, moderate differences in peak concentrations between formulations occurred. Subsequent studies should evaluate whether prolonged administration of these formulations results in more marked, clinically meaningful differences.

## Conclusions

In summary, the present study with Luminal® vet confirmed the favorable pharmacokinetics of phenobarbital for treatment of epilepsy in dogs. The rapid absorption and long half-life are important advantages for maintenance treatment of epilepsy in this species. With respect to the anecdotal reports that seizures may occasionally recur when switching treatment from Luminal® to Phenoleptil® in epileptic dogs, only one (#3) of the 8 dogs showed clear deviations between the two dosage forms in peak concentration and AUC , which may be an indication of a clinically important between-dosage form variability. However, on average both phenobarbital formulations produced almost the same plasma levels in dogs and had an almost identical relative bioavailability. Thus, the reasons for the anecdotal findings, if true, need to be further explored.

## Abbreviations

AUC: Area under the curve; Cmax: Maximal plasma concentration; HPLC: High performance liquid chromatography; t0.5: Elimination half life; Tmax: Time of maximal plasma concentration.

## Competing interests

None of the authors of this paper has a financial or personal relationship with other people or organisations that could inappropriately influence or bias the content of the paper.

## Authors’ contributions

All authors conceived and designed the study. MB and JPB performed the study and analyzed the data. All authors interpreted the data. WL wrote the paper. All authors read and approved the final manuscript.
